# Active Neutralizing Mats for Corrosive Chemical Storage

**DOI:** 10.3390/gels8080489

**Published:** 2022-08-06

**Authors:** Rui D. V. Fernandes, Liliana Melro, Jorge Padrão, Ana Isabel Ribeiro, Behnaz Mehravani, Filipa Monteiro, Eduardo Pereira, Marcos S. Martins, Nuno Dourado, Andrea Zille

**Affiliations:** 1 Centre for Textile Science and Technology (2C2T), University of Minho, 4800-058 Guimarães, Portugal; 2 ISISE, Institute of Science and Innovation in Structural Engineering, Department of Civil Engineering, University of Minho, 4800-058 Guimarães, Portugal; 3 CMEMS-UMINHO, Universidade do Minho, 4800-058 Guimarães, Portugal; 4 LABBELS—Associate Laboratory, Braga, 4800-058 Guimarães, Portugal

**Keywords:** neutralizing mats, chemical storage, composite mats, alginate, sodium carbonate, citric acid

## Abstract

Laboratories and industries that handle chemicals are ubiquitously prone to leakages. These may occur in storage rooms, cabinets or even in temporary locations, such as workbenches and shelves. A relevant number of these chemicals are corrosive, thus commercial products already exist to prevent material damage and injuries. One strategy consists of the use of absorbing mats, where few display neutralizing properties, and even less a controlled neutralization. Nevertheless, to the authors’ knowledge, the commercially available neutralizing mats are solely dedicated to neutralizing acid or alkali solutions, never both. Therefore, this work describes the development and proof of a completely novel concept, where a dual component active mat (DCAM) is able to perform a controlled simultaneous neutralization of acid and alkali leakages by using microencapsulated active components. Moreover, its active components comprise food-grade ingredients, embedded in nonwoven polypropylene. The acid neutralizing mats contain sodium carbonate (Na_2_CO_3_) encapsulated in sodium alginate microcapsules (MC-ASC). Alkali neutralizing mats possess commercial encapsulated citric acid in hydrogenated palm oil (MIRCAP CT 85-H). A DCAM encompasses both MC-ASC and MIRCAP CT 85-H and was able to neutralize solutions up to 10% (*v*/*v*) of hydrochloric acid (HCl) and sodium hydroxide (NaOH). The efficacy of the neutralization was assessed by direct titration and using pH strip measurement tests to simulate the leakages. Due to the complexity of neutralization efficacy evaluation based solely on pH value, a thorough conductivity study was performed. DCAM reduced the conductivity of HCl and NaOH (1% and 2% (*v*/*v*)) in over 70%. The composites were characterized by scanning electron microscopy (SEM), differential calorimetry (DSC) and thermogravimetric analysis (TGA). The size of MC-ASC microcapsules ranged from 2 μm to 8 μm. Finally, all mat components displayed thermal stability above 150 °C.

## 1. Introduction

Leakages in chemical storage rooms may be pragmatically considered as inevitable. Corrosive chemicals leakages promptly corrode shelves and cabinets, and simultaneously produce harmful vapors or fumes. Once the structural integrity of the shelves or the chemical cabinets is seriously compromised, they become prone to grievous accidents such as major chemical spills, fire or explosions [1,2]. The addition of chemicals to promote neutralization is a common procedure when leakage is noticed. However, the operation is risky due to the amount of heat released during neutralization. The temperature can abruptly increase, causing the evaporation of acidic or alkaline fumes. Another strategy is the use of sorbents to decrease the release of heat and minimize water consumption [3,4]. There are several commercial mats indicated for chemical storage cabinets; nevertheless, these products are prevalently focused solely on absorption, rather than on their neutralization. The most common solutions for chemical spillage in the market comprise: (i). Chemical Absorbent Pads and Rolls from First Mats Ltd. [5], (ii). Hazchem Absorbent Pads [6], (iii). Fisherbrand™ Universal All-Purpose Absorbent Pads [7], and (iv). PIG^®^ Essentials Chemical Mat Roll MATE912 [8]. Chemical spillage containment is the main purpose of these absorbent pads and rolls, being effective against most acids, alkali, coolants, paints, solvents, oils, and hazardous chemicals. However, specific acid or alkali neutralizing mats are scarce. Furthermore, eclectic neutralizing mats able to simultaneously cope with acid and alkali spills are, as far as the authors know, inexistent. The same goes for pads and rolls able to perform a controlled neutralization to mitigate exothermic reactions.

**Figure 1:**
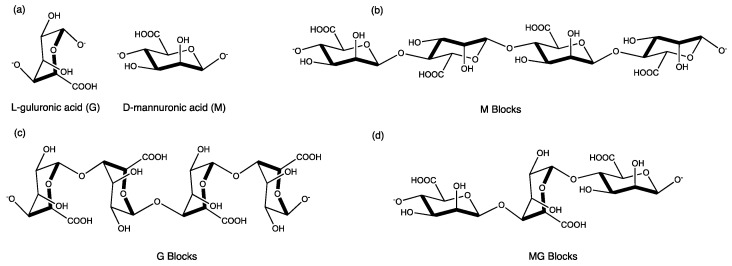
(**a**) Alginate monomers L-guluronic acid (G) and D-mannuronic acid (M) and characteristic polymeric (**b**) M blocks, (**c**) G blocks and (**d**) MG blocks [adapted from [10]]. Figure created using ChemDraw 15.1.

Neutralizing mats can assist in disabling strong acids and alkali, avoiding the vapors from diffusing into the storage room in combination with the appropriate ventilation of the cabinets, and preventing the spread of the chemicals during extensive spillage. To ensure a controlled neutralization, the neutralizing agents can be embedded in a matrix, such as alginate. Using a matrix the accumulation of hazardous vapors and exothermic overheating can be prevented as the active neutralizer to prevent its prompt exhaustion [9]. Alginate is a natural polysaccharide extracted from brown seaweed [10,11] constituted by two monomers, L-guluronic acid (G) and D-mannuronic acid (M) (Figure 1a). Depending on the seaweed species, the sequences of M and G residues (Figure 1d) may display different proportions, or, just polymeric blocks of either G or M monomers (Figure 1b,c).

**Figure 2:**
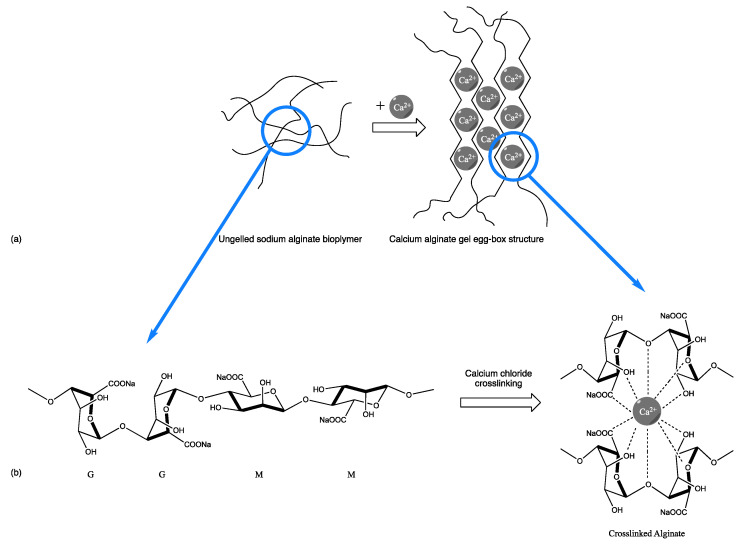
Alginates polycations crosslinking, (**a**) molecular overview, (**b**) organization at atomic level. [Adapted with permission from Ref. [20]. 2022, Elsevier].

Alginates can be found commercially in the form of sodium, ammonium, or potassium salts. Due to alginates thickening, emulsion stabilizer, biocompatible, biodegradable, non-toxic and film-forming properties, it is commonly used in several industries such as the food industry [12], biomedical industry [13,14,15,16] and pharmaceutical industry [17,18,19]. A notable example is the use of alginates, commonly used in drugs for the treatment of heartburn and indigestion (Gaviscon^®^), in combination with weak alkali, namely calcium carbonate and sodium bicarbonate, as antacid components of the formulation [12,17]. The alginate forms a viscous layer when in contact with gastric acid, impeding the reflux of gastric materials to the esophagus, while the antacid components neutralize the intragastric acids. Moreover, alginates form hydrogel beads when crosslinked with polycations [20] (being calcium chloride, one of the most used crosslinkers in alginate gelation), where the polycation forms a chelating center between alginate polymeric chains (Figure 2). This gelation method is advantageous as it can be carried out at room temperature at a neutral pH and delays the release of the encapsulated materials, as the formed gel is water-insoluble [21].

In a similar process, in which weak alkali neutralizes strong stomach acids, weak acids can be used to neutralize strong alkali solutions. Once citric acid comes into contact with alkali solutions, citrate salt and water are formed. Moreover, citrates are commonly used as buffer agents due to citric acid stability. Therefore, citric acid is vastly used in the food industry as an acidulant, stabilizer, pH regulating agent and antioxidant. In addition, due to its biocompatibility and biodegradability, citric acid is used by the pharmaceutical industry as an anticoagulant [22]. Citric acid is often encapsulated in a matrix of hydrogenated palm oil that provides improved stability [23,24].

Commercial neutralizing mats, despite being scarce, are already available. However, the development of a dual neutralizing component active mat (DCAM) that would be effective in neutralizing both acids and alkali could be advantageous in several scenarios, particularly to contain laboratory or industry transient storage areas such as a workbench, which normally comprises the simultaneous presence of both acid and alkali solutions. In addition, during accidental spills, the use of DCAM would absorb and neutralize the spilled solution, independently of its pH, even when the nature of spillage is unknown. Moreover, the use of microcapsules will be able to control the release of the active agent and avoid abrupt reactions of neutralization.

Therefore, a novel active neutralizing mat DCAM was envisioned to adequately neutralize corrosive chemicals and prevent the build-up of dangerous vapors. This work thoroughly characterizes a novel mat that encompasses in its formulation both acid neutralizing (sodium carbonate) and alkali neutralizing (citric acid) microencapsulated components. These food-grade components meet the extensive list of safety regulations that chemical storage rooms demand according to the Health and Safety Executive [25].

Therefore, the main novelty of this work was the development of a new mat that is effectively able to neutralize, simultaneously, acids and alkali spills (in distinct areas, when placed under the flasks in their storage) or during leakage control. Moreover, the mats presented in this work allow a slow and controlled neutralization reaction due to the microencapsulation of the active agents, avoiding contact with toxic fumes together with area ventilation, as well as abrupt exothermic reactions. To the authors’ knowledge, there are no commercial mats nor studies combining microcapsules for both acid and alkali neutralization in their formulation. All this is achieved with simple and fast methods and by using safe chemicals. The neutralization of acidic and alkaline solutions was assessed by direct titration, pH strip measurement and conductivity tests. The structural analysis of the composites was evaluated by scanning electron microscopy (SEM), and the thermal properties by differential calorimetry (DSC) and thermogravimetry (TGA).

This DCAM is envisaged to be used in both laboratory and chemical storage rooms, comprising cost-effective and safe materials able to prevent structural damage and reduce the risk of environmental and health hazards.

## 2. Results and Discussion

### 2.1. Evaluation of Neutralizing Efficiency

#### 2.1.1. Direct Titrations

Firstly, direct titrations were performed to observe the efficacy of microencapsulation in the neutralization process by comparison with non-encapsulated citric acid and sodium carbonate. Table 1 denotes the required mass of each compound non- and microencapsulated to neutralize 25 mL of an alkali or acid solution (1%, 2% or 10% (*v*/*v*)). MIRCAP CT 85-H corresponds to commercial encapsulated citric acid in hydrogenated palm oil. Sodium carbonate (Na_2_CO_3_) encapsulated in sodium alginate cross-linked with calcium chloride (CaCl_2_) is defined as MC-ASC. The values for MIRCAP CT 85-H are in accordance with the technical data sheet, which mentions an encapsulation percentage of 84% of citric acid in palm oil. In addition, the microcapsules MC-ASC synthesized in this work obtained an encapsulation percentage above 95%.

#### 2.1.2. PH Strip Measurement Tests of Simulated Leakage

**Figure 3:**
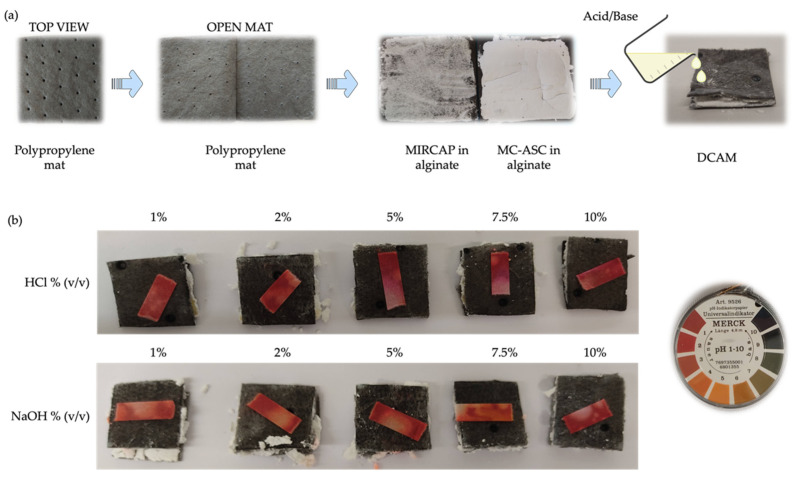
(**a**) Method for DCAM preparation; (**b**) pH strips placed on the bottom of the DCAM, exposed to the leaked acid or alkali solution at different concentrations (1 to 10% (*v*/*v*)).

A DCAM, in its formulation, comprises MIRCAP and MC-ASC embedded in an alginate matrix within a polypropylene nonwoven fabric. The surface of the developed DCAM was exposed to dropwise 1 mL of acid or alkali solution from 1 to 10% (*v*/*v*). The leaked solution was analyzed using a pH strip placed underneath the mat (Figure 3).

Figure 3b denotes that the DCAM neutralized all the tested NaOH solutions. However, the observed pH value dropped to approximately 5, probably due to the continuous release of citric acid from the MIRCAP after being exposed to the alkali solutions. The DCAM displayed a similar behavior for the HCl solutions. The exhibited acid pH value (of approximately 2) may be also due to the citric acid released from the MIRCAP.

To verify the hypothesis of the continuous release, and to ensure the adequate neutralization of both acid and alkali solutions, a thorough conductivity evaluation was performed.

#### 2.1.3. Conductivity Analysis

The conductivity is proportional to the acid or alkali strength. The stronger an acid/alkali, the greater its degree of ionization and, consequently, the greater its conductivity. A decrease in conductivity will reflect the neutralization reaction [26]. Thus, using either strong acids or strong alkalis will only vary the area of the DCAM needed to neutralize them. Stronger acids/alkali will need more area and weaker acids/alkali will need less area when the same amount and concentration of acid/alkali are spilt. In this work, HCl and NaOH (1% and 2% (*v*/*v*)) were used as an example to prove the neutralizing effect. 

**Figure 4:**
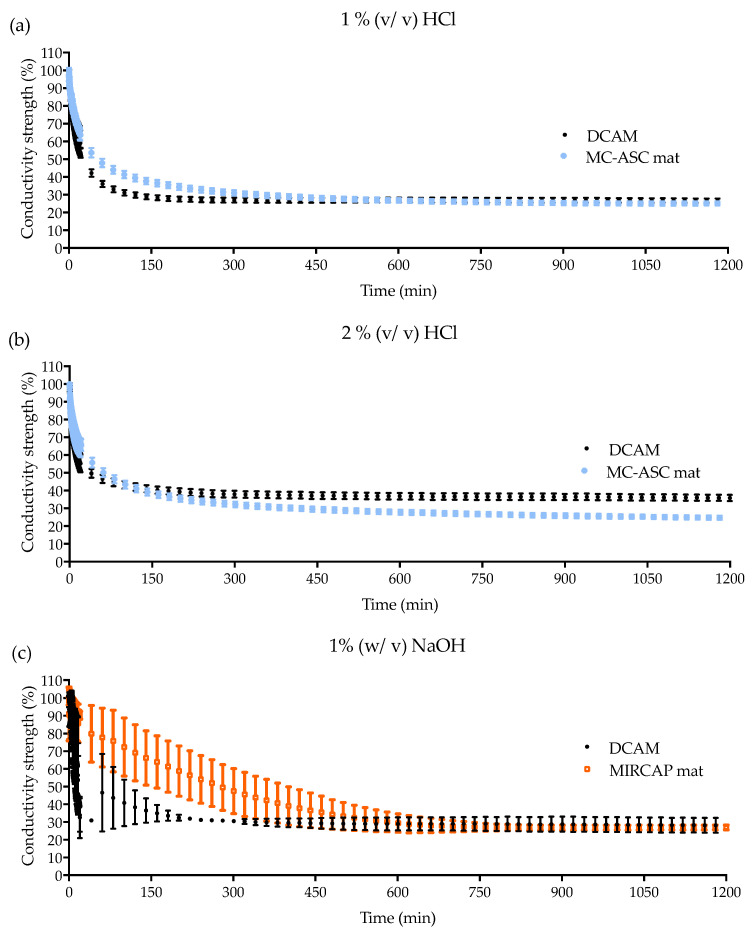

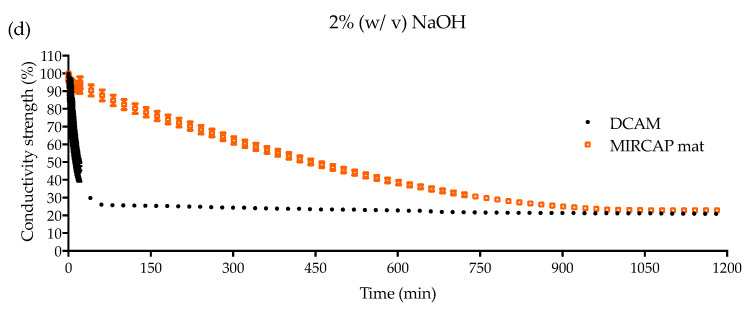
Conductivity profiles of solutions in contact with DCAM in: (**a**) HCl 1% (*v*/*v*), (**b**) HCl 2% (*v*/*v*), (**c**) NaOH 1% (*v*/*v*), and (**d**) NaOH 2% (*v*/*v*) (the results represent the average of the conductivity of triplicate test during independent assays. All samples displayed a maximum standard deviation (SD) of ±5%, except samples tested in NaOH 1% which presented a SD of ±12%).

The conductivity of the solutions (HCl or NaOH) was measured over a period of 20 h in the presence of each neutralizing mat. The obtained conductivity curves are presented in Figure 4. Considering that distilled water has nearly no conductivity, with the immersion of the alkali neutralizing mat containing the MIRCAP CT 85-H microcapsules embedded in alginate, the conductivity increased to a maximum of 1.6 mS·cm ^−1^ in the first 15 min, denoting an increase in electrolytes in the solution. On the contrary, the acid neutralizing mat containing a mixture of sodium carbonate in alginate showed a small increase in conductivity to a maximum of 102.4 µS·cm^−1^ in the first 15 min. These results are expected since the citric acid in water promotes the presence of H_3_O^+^ ions, whereas the sodium carbonate does not promote HO^−^ ions [26,27]. The DCAM showed a behavior intermediate to both acid and alkali neutralizing mats, with a conductivity of 941.8 µS·cm^−1^ in the first 15 min.

The conductivity of HCl and NaOH solutions of 1% (*v*/*v*) and 2% (*v*/*v*) were also tested in the presence of acid and alkali neutralizer mats, and DCAM. Figure 4a,b shows the conductivity of HCl solutions of 1% (*v*/*v*) and 2% (*v*/*v*), respectively. The 1% (*v*/*v*) HCl solution required a 9 cm^2^ mat to reach a neutral pH whilst the 2% (*v*/*v*) required an area of 18 cm^2^. For both solutions, the acid neutralizing and the DCAM denote a decrease in conductivity of approximately 40% after 15 min. Therefore, the neutralization of HCl at 2% (*v*/*v*) is equivalent to the initial conductivity of the HCl solution at 1% (*v*/*v*). After 20 h both 1 and 2% HCl solutions had a loss of approximately 75% in conductivity.

Regarding the DCAM neutralization of NaOH (Figure 4c,d), a similar behavior was observed for NaOH 1% (*v*/*v*) solution, where MIRCAP CT 85-H neutralizer mat had a similar conductivity loss as the DCAM. However, for NaOH 2% (*v*/*v*), the decrease in conductivity was sharper in DCAM in comparison to MIRCAP CT 85-H mat. To understand this behavior, conductivity tests with MIRCAP CT 85-H without the alginate matrix involving it were performed. Conductivity of NaOH 1% (*v*/*v*) solution had a decay of 30% while NaOH 2% solution decreased by 43%. Considering these results, it was concluded that the alginate matrix delays the release of MIRCAP CT 85-H, resulting in a slower response. This delay is longer as the concentration of NaOH rises, since alginate solubility decreases inversely to pH value. This delay may be the main reason for the higher standard deviations observed in NaOH.

Measurements performed in the solutions with the mats after 20 h of immersion showed a decrease in conductivity of approximately 75% for both 1 and 2% NaOH solutions. Therefore, the successful neutralization of both acid an alkali was achieved.

### 2.2. Scanning Electron Microscopy (SEM) Analysis

**Figure 5:**
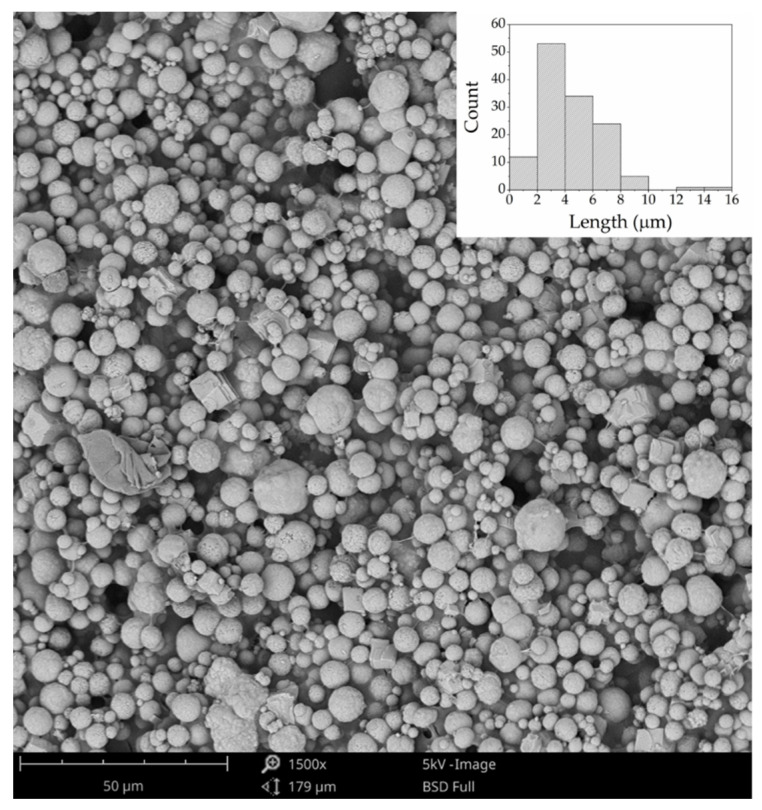
SEM image of MC-ASC deposited inside the mat. The histogram inset represents the size distribution of MC-ASC beads.

The structural analysis of the mat containing the MC-ASC can be visualized in the micrographs in Figure 5. The image showed a well-distribution of the microcapsules onto the mat, quasi-spherical shape microcapsules, and some variations in the MC-ASC size that ranged mostly from 2 to 8 μm. The microencapsulation successfully entrapped the active component to avoid abrupt chemical reactions and without compromising the neutralization efficacy.

### 2.3. Thermal Analysis

**Figure 6:**
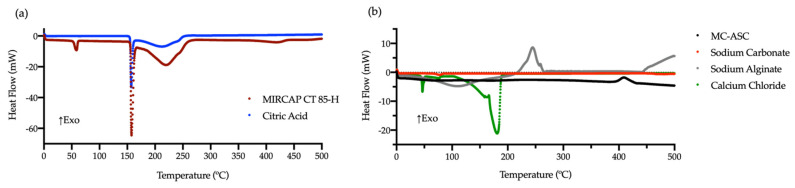
DSC curve of citric acid and hydrogenated palm oil encapsulated citric acid (MIRCAP CP 85-H) (**a**), and of all compounds used in the synthesis of acid neutralizer MC-ASC (**b**).

**Figure 7:**
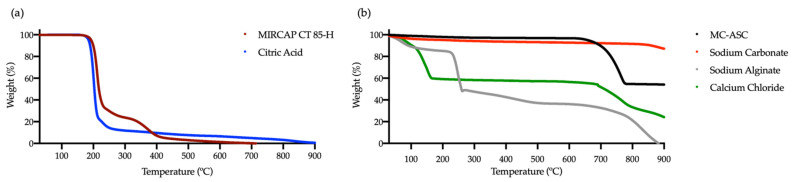
TGA curves of citric acid and hydrogenated palm oil encapsulated citric acid (MIRCAP CP 85-H) (**a**), and of all compounds used in the synthesis acid neutralizer MC-ASC (**b**).

The differential scanning calorimetry (DSC) and thermogravimetry (TGA) curves of all compounds are shown in Figure 6 and Figure 7. Analyses were performed at a heating rate of 10 °C.min^−1^ in an alumina crucible and with a nitrogen flow rate of 200 mL.min^−1^. The DSC curves for MC-ASC microcapsules (Figure 6) differ from the other components used in the synthesis, confirming the successful microencapsulation of sodium carbonate. The DSC and TGA curves of CaCl_2_ [28,29,30] were very high, since it only has a total weight loss of 14.1% at 920 °C [31].

MC-ASC presents a glass transition temperature (Tg) at 61.6 °C and an endothermic peak, with enthalpy energy of 26.5 J.g^−1^, at 409.4 °C. TGA curves of MC-ASC (Figure 7) showed that the DSC endothermic peak was not associated with weight loss, meaning that it can be attributed to a structural rearrangement of the microcapsules to a more stable form, typical of a curing process. The major thermal transitions of all compounds are presented in Table 2, including the thermal behavior of MIRCAP CT 85-H. When compared to pure citric acid [32], MIRCAP CT 85-H DSC curve presents two extra peaks, at 58.6 °C and 420.9 °C, attributed to the melting and decomposition, respectively, of hydrogenated palm oil used in the encapsulation process. It was also observed that due to the presence of hydrogenated palm oil, the citric acid decomposition of MIRCAP CT 85-H started approximately 10 °C above the decomposition temperature of pure citric acid. The energy necessary to decompose citric acid in MIRCAP CT 85-H was lower, 387.9 J.g^−1^, than the pure citric acid, 449.5 J.g^−1^. These values can be explained by the encapsulation percentage of citric acid in MIRCAP CT 85-H (84.4%).

According to TGA curves (Figure 7), MC-ASC microcapsules proved to be thermally stable until 644 °C, a temperature from which the microcapsules began to decompose until stabilizing at 781 °C, with a weight loss of 42% was attributed to the decomposition of the crosslinked calcium alginate. As the melting point of the carbonate was around 850 °C [33] and no large weight loss was observed in either TGA curves, it is assumed that the residual weight of 53.9% of the MC-ASC at 920 °C is the encapsulated sodium carbonate, as its decomposition temperature was above the temperature capacity of the STA apparatus used for these experiments.

Regarding MIRCAP CT 85-H, the TGA curve presented two major thermal changes. The first appeared at temperatures between 160 and 296 °C, which is attributed to the thermal decomposition of citric acid (weight loss of 76.0%) [34]. These data are supported by the pure citric acid TGA curve, where the decomposition temperature is between 160 and 293 °C. The second thermal change is set between temperatures of 296 and 456 °C, attributed to the decomposition of the encapsulating material: hydrogenated palm oil, which represented a weight loss of 20%.

## 3. Conclusions

Mats combining microencapsulated active components to neutralize both acid and alkali were developed for the first time. The DCAM displays an impressive ability of neutralization, through a controlled release of the active agents. This controlled release represents a critical feature, since when a chemical leakage occurs, an uncontrolled reaction between the chemicals may unfold into a dangerous exothermic and hazardous fume-generating event. To the authors’ knowledge, the developed DCAM possesses a completely novel and unique feature of being able to simultaneously neutralize acid and alkali leakages. Furthermore, it displays superior neutralization effectiveness. The DCAM was developed to neutralize possible leaks of chemicals that are unattended for long periods of time or cleaning small/medium spillages. The use of controlled, effective and eclectic neutralizing mats is an important step forward in improving the safety of chemical storage and working environments worldwide. 

## 4. Materials and Methods

### 4.1. Materials

Citric acid (C_6_H_8_O_7_, Scharlau, Barcelona, Spain) was used as the neutralizing agent for alkali solutions and sodium carbonate (Na_2_CO_3_, Scharlau, Barcelona, Spain) was used as an acid neutralizer. Calcium chloride dihydrate (CaCl_2_ 2H_2_O, Merck, Darmstadt, Germany) served as an ionic gelling agent when forming microspheres of sodium carbonate containing sodium alginate at 1 wt%, ((C_6_H_7_O_6_Na)_n_, M = 10,000–600,000 g/mol, PanReac AppliChem ITW Reagents, Barcelona, Spain). Microcapsules of citric acid encapsulated in hydrogenated palm oil (84.4% (*w*/*w*), 509.6 ± 167.0 µm) (MIRCAP CT 85-H) were kindly supplied by VEDEQSA (Venta de Especialidades Químicas, S.A., Barcelona, Spain). Alkali solutions were prepared using sodium hydroxide (NaOH, pellets, Merck, Darmstadt, Germany). Acid solutions were prepared using hydrochloric acid (HCl 37% (*v*/*v*), Merck, Darmstadt, Germany). Polypropylene Absorbent Mat Pad MAT 412 was used as substrate (thickness of 4.37 ± 0.17 mm).

### 4.2. Methods

#### 4.2.1. Direct Titrations

In order to estimate the amount of citric acid and sodium carbonate needed to neutralize the alkali or acid solutions, and therefore the quantity to incorporate into the mats, titrations were performed in 25 mL of 1%, 2% and 10% (*v*/*v*) of NaOH or HCl. For conductivity studies, only solutions of 1% and 2% (*v*/*v*) were performed due to the linearity of the results.

#### 4.2.2. Microcapsules Production

**Figure 8:**
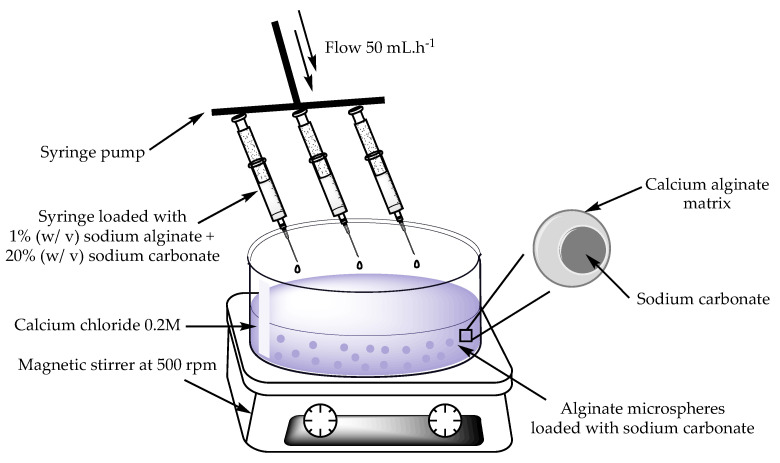
Schematic representation of the microencapsulation of sodium carbonate in sodium alginate. Figure generated using ChemDraw 15.1.

Considering the importance of a controlled release of the compounds for a safe neutralization, the latter were encapsulated prior to being introduced in the mats. Sodium carbonate microspheres were obtained using a syringe pump (NE-1600, New Era Pump Systems, Norleq, Santo Tirso, Portugal). A solution of 1% (*w*/*v*) of sodium alginate was mixed with sodium carbonate powder at 20% (*w*/*v*). The resulting mixture was then loaded into 25 mL syringes coupled to the pump and then added dropwise using a 25-gauge (G) needle into a coagulation bath of a CaCl_2_ (0.2 M) aqueous solution at a rate of 50 mL.h^−1^. The microspheres were then vacuum filtered at 1 bar, thoroughly washed with distilled water to remove excess CaCl_2_ and dried at 40 °C overnight (Figure 8).

#### 4.2.3. Scanning Electron Microscope (SEM)

Microscopy imaging was obtained using a desktop SEM coupled with energy dispersive X-ray spectroscopy (EDS) analysis (Phenom ProX with EDS detector (Phenom-World BV, Eindhoven, The Netherlands)).

#### 4.2.4. Neutralizing Mats Preparation

The microcapsules were mixed in a 1% (*w*/*v*) sodium alginate solution and added to a mat with an area of 156.25 cm^2^. The encapsulated neutralizer mixture was spread onto one side of the mat. The other side was placed on top and enclosed with spray adhesive Foam Fast 74 from 3 M, followed by padding at 0.1 bar and a velocity rate of 4 m/min on a Padder BHP (Roaches). For the DCAM, one side of the mat was covered with the mixture containing MC-ASC and the other with MIRCAP.

#### 4.2.5. Conductivity and PH

Conductivity was measured using a Thermo Scientific Orion Versa Star Pro Advanced Electrochemistry Meter. Pre-tests were performed to observe if the results of the conductivity were directly proportional in acidic or alkaline solutions from 1 to 10% concentrated solutions. Since this condition has been verified, only 1% and 2% concentrations were used for the conductivity tests. Mats with an area of 9 cm^2^ were immersed in alkali or acid solutions of NaOH and HCl. For the 1% (*v*/*v*) solutions, a single mat was immersed, whilst for the 2% (*v*/*v*) solutions two mats were required (18 cm^2^). Conductivity was measured over a period of 20 h with reading intervals of 5 s in the first 20 min, and in intervals of 20 min until 20 h. pH measurements were performed using a Thermo Scientific Orion Versa Star Pro Advanced Electrochemistry Meter and pH-indicator paper (Merck).

#### 4.2.6. Thermal Analysis

DSC measurements were performed in a Mettler Toledo DSC 822e apparatus (Mettler Toledo, Columbus, OH, USA). The temperature range was between 20 °C and 500 °C at a heating rate of 10 °C·min^−1^ in an alumina crucible and with a nitrogen flow rate of 200 mL.min^−1^. The initial mass was measured with a digital fully automatic Mettler Toledo calibration balance (AB 204-S/FACT Classic Plus, Mettler Toledo, Columbus, OH, USA). DSC curves were plotted with heat flow versus temperature.

Thermogravimetric analyses (TGA) were carried out in a STA 7200 Hitachi^®^ (Fukuoka, Japan). TGA and differential thermal analyses (DTA) are shown simultaneously. TGA profiles were obtained within the range of 20 to 920 °C under a nitrogen atmosphere (200 mL.min^−1^) at 10 °C.min^−1^. The derivative thermogravimetric (DTG) analysis was performed to identify the thermal transformation events (namely the maximum peaks).

## Figures and Tables

**Table 1:** Compounds mass needed for complete neutralization of 25 mL of hydrochloric acid (HCl) and sodium hydroxide (NaOH).

	Citric Acid (g)	MIRCAP CT 85-H (g)	Na_2_CO_3_ (g)	MC-ASC (g)
HCl (% (*v*/*v*))				
1	-	-	0.459 ± 0.004	0.475 ± 0.043
2	-	-	0.891 ± 0.010	0.946 ± 0.073
10	-	-	4.321 ± 0.025	4.413 ± 0.204
NaOH (% (*v*/*v*))				
1	0.419 ± 0.004	0.505 ± 0.004	-	-
2	0.788 ± 0.024	0.954 ± 0.054	-	-
10	3.922 ± 0.055	4.639 ± 0.136	-	-

**Table 2:** DSC and TGA values of the compounds used in the production of neutralizing mats.

	DSC				TGA		
	Temperature (°C)	Enthalpy (J.g^−1^)	DTG Peak (°C)	T_onset_ (°C)	T_offset_ (°C)	Weight Loss (%)	Residual Weight at 920 °C (%)
**CaCl_2_.2H_2_O**	46.7	−26.8	49.1	49.0	102.5	10	19.5
	180.9	−855.6	140.1; 154.3	102.9	168.2	30	
			768.9	692.8	914.8	33	
**Sodium Alginate**	107.8	−233.6	77.5	28.2	160.8	14	0
	244.8	183.1	246.2	233.9	262.0	32	
			842.8	605.5	920.0	45	
**Na_2_CO_3_**	77.1	−12.4	76.4	23.9	860.0	10	85.9
	356.0	T_g_	888.2	861.1	920.0	4	
**MC-ASC**	61.6	T_g_	764.9	25.5	644.9	4	53.9
	409.4	26.5		646.2	780.1	42	
**Citric Acid**	157.2	−200.6	201.3	163.8	292.2	88	0.3
	214.5	−449.5					
**MIRCAP CT 85-H**	58.6	−24.3	211.9	176.6	312.5	76	0
	157.6	−177.0					
	221.0	−387.9	375.1	312.5	456.6	20	
	420.9	−21.0					

## References

[1] Asiry S., Ang L.-C. (2019). Laboratory Safety: Chemical and Physical Hazards. Biobanking.

[2] Zhang C. (2018). Analysis of Fire Safety System for Storage Enterprises of Dangerous Chemicals. Procedia Eng..

[3] Vergara-Rubio A., Ribba L., Picón D., Candal R., Goyanes S. (2022). A Highly Efficient Nanostructured Sorbent of Sulfuric Acid from Ecofriendly Electrospun Poly(vinyl alcohol) Mats. Ind. Eng. Chem. Res..

[4] Zaffora A., Culcasi A., Gurreri L., Cosenza A., Tamburini A., Santamaria M., Micale G. (2020). Energy Harvesting by Waste Acid/Base Neutralization via Bipolar Membrane Reverse Electrodialysis. Energies.

[5] First Mats Ltd. https://www.firstmats.co.uk/.

[6] Global Spill Australia. https://www.globalspill.com.au.

[7] Fisher Scientific UK Ltd. https://www.fishersci.co.uk.

[8] New Pig, UK. https://www.newpig.eu/.

[9] Choi H., Song Y.K., Kim K.Y., Park J.M. (2012). Encapsulation of triethanolamine as organic corrosion inhibitor into nanoparticles and its active corrosion protection for steel sheets. Surf. Coat. Technol..

[10] Ching S.H., Bansal N., Bhandari B. (2015). Alginate gel particles—A review of production techniques and physical properties. Crit. Rev. Food Sci. Nutr..

[11] Fernandes M., Padrão J., Ribeiro A.I., Fernandes R.D.V., Melro L., Nicolau T., Mehravani B., Alves C., Rodrigues R., Zille A. (2022). Polysaccharides and Metal Nanoparticles for Functional Textiles: A Review. Nanomaterials.

[12] Smith A.M., Miri T. (2010). Alginates in Foods. Practical Food Rheology.

[13] Venkatesan J., Bhatnagar I., Manivasagan P., Kang K.-H., Kim S.-K. (2015). Alginate composites for bone tissue engineering: A review. Int. J. Biol. Macromol..

[14] Ahmad A., Mubarak N.M., Jannat F.T., Ashfaq T., Santulli C., Rizwan M., Najda A., Bin-Jumah M., Abdel-Daim M.M., Hussain S. (2021). A Critical Review on the Synthesis of Natural Sodium Alginate Based Composite Materials: An Innovative Biological Polymer for Biomedical Delivery Applications. Processes.

[15] Chen K., Wang F., Liu S., Wu X., Xu L., Zhang D. (2020). In situ reduction of silver nanoparticles by sodium alginate to obtain silver-loaded composite wound dressing with enhanced mechanical and antimicrobial property. Int. J. Biol. Macromol..

[16] Ma R., Wang Y., Qi H., Shi C., Wei G., Xiao L., Huang Z., Liu S., Yu H., Teng C. (2019). Nanocomposite sponges of sodium alginate/graphene oxide/polyvinyl alcohol as potential wound dressing: In vitro and in vivo evaluation. Compos. Part B Eng..

[17] Wilkinson J., Abd-Elaziz K., den Daas I., Wemer J., van Haastert M., Hodgkinson V., Foster M., Coyle C. (2018). Two placebo-controlled crossover studies in healthy subjects to evaluate gastric acid neutralization by an alginate–antacid formulation (Gaviscon Double Action). Drug Dev. Ind. Pharm..

[18] Afshar M., Dini G., Vaezifar S., Mehdikhani M., Movahedi B. (2020). Preparation and characterization of sodium alginate/polyvinyl alcohol hydrogel containing drug-loaded chitosan nanoparticles as a drug delivery system. J. Drug Deliv. Sci. Technol..

[19] Yang J.-S., Xie Y.-J., He W. (2011). Research progress on chemical modification of alginate: A review. Carbohydr. Polym..

[20] Gao X., Guo C., Hao J., Zhao Z., Long H., Li M. (2020). Adsorption of heavy metal ions by sodium alginate based adsorbent-a review and new perspectives. Int. J. Biol. Macromol..

[21] Sachan N., Pushkar S., Jha A., Bhattcharya A. (2009). Sodium alginate: The wonder polymer for controlled drug delivery. J. Pharm. Res..

[22] Apelblat A. (2014). Citric Acid.

[23] VEDEQSA https://www.vedeqsa.com/.

[24] Watson Inc. https://www.watson-inc.com/product/citric-acid-85-palm/.

[25] HSE—Health and Safety Executive (2009). Chemical Warehousing: The Storage of Packaged Dangerous Substances.

[26] Artemov V.G., Volkov A.A., Sysoev N.N., Volkov A.A. (2015). Conductivity of aqueous HCl, NaOH and NaCl solutions: Is water just a substrate?. EPL Europhys. Lett..

[27] Munjal S., Singh A. (2019). The Arrhenius Acid and Base Theory.

[28] Gaeini M., Rouws A.L., Salari J.W.O., Zondag H.A., Rindt C.C.M. (2018). Characterization of microencapsulated and impregnated porous host materials based on calcium chloride for thermochemical energy storage. Appl. Energy.

[29] Karunadasa K.S.P., Manoratne C.H., Pitawala H.M.T.G.A., Rajapakse R.M.G. (2018). Relative stability of hydrated/anhydrous products of calcium chloride during complete dehydration as examined by high-temperature X-ray powder diffraction. J. Phys. Chem. Solids.

[30] Fraissler G., Jöller M., Brunner T., Obernberger I. (2009). Influence of dry and humid gaseous atmosphere on the thermal decomposition of calcium chloride and its impact on the remove of heavy metals by chlorination. Chem. Eng. Process. Process Intensif..

[31] Newkirk A.E., Aliferis I. (2002). Drying and Decomposition of Sodium Carbonate. Anal. Chem..

[32] Gumieniczek A., Trębacz H., Komsta Ł., Atras A., Jopa B., Szumiło M., Popiołek Ł. (2018). DSC, FT-IR, NIR, NIR-PCA and NIR-ANOVA for determination of chemical stability of diuretic drugs: Impact of excipients. Open Chem..

[33] Harris J.D., Rusch A.W. (2012). Identifying Hydrated Salts Using Simultaneous Thermogravimetric Analysis and Differential Scanning Calorimetry. J. Chem. Educ..

[34] Wyrzykowski D., Hebanowska E., Nowak-Wiczk G., Makowski M., Chmurzyński L. (2010). Thermal behaviour of citric acid and isomeric aconitic acids. J. Therm. Anal. Calorim..

